# Discovery of a Novel and Rich Source of Gluten-Degrading Microbial Enzymes in the Oral Cavity

**DOI:** 10.1371/journal.pone.0013264

**Published:** 2010-10-11

**Authors:** Eva J. Helmerhorst, Maram Zamakhchari, Detlef Schuppan, Frank G. Oppenheim

**Affiliations:** 1 Department of Periodontology and Oral Biology, Boston University Henry M. Goldman School of Dental Medicine, Boston, Massachusetts, United States of America; 2 Division of Gastroenterology, Beth Israel Deaconess Medical Center, Harvard Medical School, Boston, Massachusetts, United States of America; National Institute of Allergy and Infectious Diseases, National Institutes of Health, United States of America

## Abstract

**Background:**

Celiac disease is a T cell mediated-inflammatory enteropathy caused by the ingestion of gluten in genetically predisposed individuals carrying HLA-DQ2 or HLA-DQ8. The immunogenic gliadin epitopes, containing multiple glutamine and proline residues, are largely resistant to degradation by gastric and intestinal proteases. Salivary microorganisms however exhibit glutamine endoprotease activity, discovered towards glutamine- and proline-rich salivary proteins. The aim was to explore if gliadins can serve as substrates for oral microbial enzymes.

**Methodology/Principal Findings:**

Proteolytic activity in suspended dental plaque was studied towards a) gliadin-derived paranitroanilide(pNA)-linked synthetic enzyme substrates b) a mixture of natural gliadins and c) synthetic highly immunogenic gliadin peptides (33-mer of α2-gliadin and 26-mer of γ-gliadin). In addition, gliadin zymography was conducted to obtain the approximate molecular weights and pH activity profiles of the gliadin-degrading oral enzymes and liquid iso-electric focusing was performed to establish overall enzyme iso-electric points. Plaque bacteria efficiently hydrolyzed Z-YPQ-pNA, Z-QQP-pNA, Z-PPF-pNA and Z-PFP-pNA, with Z-YPQ-pNA being most rapidly cleaved. Gliadin immunogenic domains were extensively degraded in the presence of oral bacteria. Gliadin zymography revealed that prominent enzymes exhibit molecular weights >70 kD and are active over a broad pH range from 3 to 10. Liquid iso-electric focusing indicated that most gliadin-degrading enzymes are acidic in nature with iso-electric points between 2.5 and 4.0.

**Conclusions/Significance:**

This is the first reported evidence for gluten-degrading microorganisms associated with the upper gastro-intestinal tract. Such microorganisms may play a hitherto unappreciated role in the digestion of dietary gluten and thus protection from celiac disease in subjects at risk.

## Introduction

The gastrointestinal (GI) tract consists of distinct but connected anatomical regions, comprising the oral cavity, the oesophagus, the stomach, the small and the large intestine[1]. The entire GI tract is colonized with microorganisms with colonization levels showing a gradient being lowest in the stomach and increasing in density toward the proximal and distal ends. The proximal region, the oral cavity, provides a rich environment for bacterial colonization as it contains a variety of different habitats and ecological niches. It harbors over 600 different types of bacteria belonging to 141 different taxa representing 6 different bacterial phyla, including the *Firmicutes*, *Actinobacteria*, *Proteobacteria*, *Bacteroidetes*, *Fusobacteria* and the *TM7* phylum [2], [3]. The distal GI microbiome is likewise phylogenetically diverse with members representing at least 9 different phyla [4], [5].

The GI tract is considered a “super organ” with functions contributed by human as well as bacterial genes [6], [7]. Most GI-colonizing microorganisms live in symbiosis with the host. The mutually beneficial relationship between the host and its colonizers is most evident in aspects related to digestion. Complex carbohydrates that cannot be degraded by the arsenal of human digestive enzymes can in most cases be hydrolyzed by bacterial glycosidases. For instance, bacteria belonging to the *Bacteroides* genus, turn the non-digestable polysaccharides into small chain fatty acids that are subsequently metabolized by the host [7]. While the role of GI bacteria in the metabolism of polysaccharides is being unraveled at the molecular level, the contribution of microbial enzymes to the digestion of proteins is much less thoroughly investigated. Ingested proteins are in part degraded by host proteolytic enzymes such as pepsin, elastase, carboxypeptidase, trypsin, and chymotrypsin converting them into oligopeptides and single amino acids which can then enter the enterocyte via selective transporters and be further metabolized or transported [8]. Evidently, this mechanism is contingent upon the susceptibility of the substrate proteins to digestion by host enzymes.

Examples of dietary proteins that are difficult to digest by host proteolytic enzymes are glutens. Gluten proteins, comprising gliadins and glutenins, are abundantly contained in dietary products made of wheat, barley and rye [9]. They are unusual with respect to their high content of the amino acids proline and glutamine, which are largely resistent to cleavage by the major human GI digestive enzymes. Thus pepsin or trypsin are unable to cleave the peptide bonds C-terminal to these residues [10]. The proteolytic resistance of some highly T cell stimulatory gluten-derived peptides that reach the duodenum, e.g. a 33-mer peptide from α2-gliadin and a 26-mer peptide from γ-gliadin, is paradigmatic for the inability of the human body to thoroughly digest gluten, resulting in the destructive immunological responses in the proximal intestine of patients with celiac disease [11], [12], [13].

Proteolytic degradation of protease-resistant domains in gluten appears to require enzymatic cleavage specificities that are not readily available in the repertoire of mammalian digestive enzymes. Recent studies have revealed that the oral cavity contains microorganisms producing endoprotease(s) with cleavage specificity after a glutamine residue [14]. The activity was found by analyzing the sequences of salivary peptides that are naturally present in human whole saliva. The salivary peptidome contains multiple proline- and glutamine rich peptides derived from human salivary basic proline-rich proteins that were cleaved after the Xaa-Pro-Gln tripeptide sequence[14], [15], [16]. We therefore studied the structural similarity between gliadins and basic proline-rich proteins and investigated if gliadins may be substrates for oral microbial proteases and be degraded into non-immunogenic peptides. The prospect of natural gluten degrading microorganisms associated with the oral cavity, i.e. the very proximal GI tract, would open promising new avenues in the quest to develop strategies to neutralize the deleterious effects of gluten in patients with celiac disease.

## Materials and Methods

### Dental plaque and whole saliva collection

Protocols for the collection of human dental plaque and whole saliva (WS) were approved by the Institutional Review Board at Boston University. Written informed consent was obtained from all participating subjects prior to sample collection. Plaque was obtained from a healthy subject 48 h after cessation of oral hygiene. The sample was collected from interproximal spaces using a sterile dental scaler and suspended in saliva ion buffer containing 50 mM KCl, 1.5 mM potassium phosphate, 1 mM CaCl2 and 0.1 mM MgCl2, pH 7.0 [14]. The plaque material was dispersed by pipetting and vortexing and the optical density (OD) of the resulting suspension was determined spectrophotometrically at 620 nm. Stimulated WS (5 ml) was collected from eight healthy subjects between 10.00 and 11.00 a.m. at least 1 hour after the last meal. Whole saliva flow was stimulated by mastication on four square inches of Parafilm (American National Can™, Chicago, IL). All samples were kept on ice.

### Enzyme activity analysis

Four synthetic analogs, Z-YPQ-pNA, Z-QQP-pNA, Z-PPF-pNA and Z-PFP-pNA, were chosen as representative gliadin-derived substrates and chemically synthesized (Anaspec, Fremont, CA). All substrates were dissolved in 50–75% dimethyl sulfoxide to 20 mM. From this solution, 2 µl was mixed with 200 µl plaque suspension (OD620 1.2) in saliva ion buffer or with 200 µl trypsin (final concentration 0.2 µg/ml) in 50 mM ammonium bicarbonate buffer, pH 8.0. All incubations were carried out in 96-well microtiter plates. Enzyme activity was determined from the proteolytic removal of the paranitroanilide group which was monitored spectrophotometrically at 405 nm. Z-YPQ-pNA, Z-PPF-pNA and Z-PFP-pNA showed mild precipitation upon mixing with the plaque suspension in saliva ion buffer which did not interfere with efficient substrate hydrolysis. All values were corrected for the lowest absorbance values measured after addition of the enzyme source to the substrate.

### Degradation of gliadin and gliadin peptides

A mixture of gliadins was purchased from Sigma (St. Louis, MO) and dissolved to 5 mg/ml in 60% (v/v) ethanol. Highly immunogenic peptides derived from α2-gliadin (LQLQPFPQPQLPYPQPQLPYPQPQLPYPQPQPF; 33-mer) [17] or γ-gliadin (FLQPQQPFPQQPQQPYPQQPQQPFPQ; 26-mer) [18] were chemically synthesized at a purity of 95% (21st Century Biochemicals, Marlboro, MA). Both peptides were dissolved in milliQ water at 10 mg/ml, and their concentration was verified by measurement of the OD at 215 nm applying a specific absorption coefficient ε = 20. The mixture of gliadins, the 33-mer or the 26-mer were added to a suspension of dental plaque bacteria in saliva ion buffer (OD620 1.2) or to WS to final concentrations of 250 µg/ml. After various incubation time intervals, 100 µl aliquots were removed and boiled to inactivate enzyme activity. The 100 µl gliadin-containing sample aliquots were analyzed by SDS-PAGE. The 33-mer/26-mer-containing sample aliquots were subjected to RP-HPLC.

### SDS PAGE

To the gliadin degradation sample aliquots, EDTA was added to a final concentration of 2.5 mM. Samples were dried using a speed-vac (Savant, Thermo Electron, Waltham, MA) and suspended in SDS sample buffer containing 0.4 mM EDTA, 2% (w/v) sodium dodecyl sulfate (SDS), 278 mM Tris/HCl (pH = 8.5), 292 mM sucrose, 0.075% Serva Blue G250 (w/v) and 0.025% Phenol Red (w/v). Samples were loaded onto pre-cast 12% gels (Novex, InVitrogen, Carlsbad, CA). Running buffer contained 50 mM 2-(N-morpholino) ethane sulfonic acid (MES), 50 mM Tris base (pH = 7.3), 0.1% (w/v) SDS. Electrophoresis was carried out at a constant voltage of 120 V. Gels were stained for 24 h with 0.1% Coomassie brilliant blue in 40% (v/v) methanol and 10% (v/v) acetic and destained in the same solution without dye.

### Reversed-Phase High Performance Liquid Chromatography (RP-HPLC)

To the incubation sample aliquots containing the 33-mer or the 26-mer, 900 µl 0.1% (v/v) trifluoroacetic acid (TFA) was added followed by filtration of the sample over a 0.22 µm filter (Pall Cooperation, Ann Arbor, MI). RP-HPLC of the samples was carried out using a HPLC Model 715 (Gilson, Middleton, WI) and a C-18 column (TSK-GEL 5 µm, ODS-120T, 4.6×250 mm, TOSOHaas, Montgomeryville, PA). The 33-mer, 26-mer and fragments thereof were eluted using a linear gradient from 0% to 55% buffer B containing 80% (v/v) acetonitrile and 0.1% (v/v) TFA over a 75 min time interval at a flow rate of 1.0 ml/min [19]. The eluate was monitored at 219 and 230 nm and eluting fractions were collected using peak width and peak sensitivity settings of 1.2 and 5, respectively (Unipoint version 3.3, Gilson).

### Liquid Chromatography Electrospray Ionization Tandem Mass Spectrometry (LC-ESI-MS/MS)

Mass spectrometry was conducted using a capillary nano-flow liquid chromatography and electrospray ionization tandem mass spectrometer (LC-ESI-MS/MS). HPLC fractions containing individual gliadin degradation peptides were concentrated under vacuum, suspended in 5% acetonitrile in 0.1% formic acid, and 1–3 µl samples were injected using an autosampler (Micro AS, Thermo Finnigan, San Jose, CA). Separation/elution of peptides was achieved using an in-line capillary C-18 column (Magic C-18, Micron Bioresource) applying a gradient from 5 to 95% acetonitrile in 0.1% formic acid over a 35 min time interval at a flow rate of 250 nl/min. Micro-LC separation generated the total ion (base peak) chromatogram in a survey scan with an m/z range from 400–2000. This was followed by the sequential selection of five peptide ions for collision induced dissociation in descending order of signal intensity. The process incorporated a “dynamic exclusion” step of abundant peptide ions allowing also the detection of lower abundance peptide ions within the total ion chromatogram.

The raw MS data of the mixture of gliadins were searched against a total gluten/gliadin database which was downloaded from http://appliedbioinformatics.wur.nl/. The database contained 618 gliadin and glutenin-derived protein entries from *Triticum aestivium* (wheat). The raw MS data of the fragments from the 33-mer and the 26-mer were searched against an in-house generated database containing just these two peptides. The MS/MS spectra obtained were searched using SEQUEST software (Bioworks Browser 3.3.1, Thermo-Finnigan). The SEQUEST X-corr values applied for non-tryptic peptides were as reported previously [20] and the ΔCn value was set at >0.1. Each search result was furthermore validated by manual inspection of the acquired MS/MS spectra to ensure that the fragment ions (e.g. *b* and *y* ions) were above background level and that three consecutive b- and y-ions we found for each identified peptide. Due to the highly repetitive nature of sequences within the gliadin structure and the limited variety of amino acids, data could not be searched against reversed gluten/gliadin databases as a decoy. Rather, a stringent peptide probability setting of 0.05 was applied. To establish the suitability of the selected setting to avoid false positive identifications, MS/MS data of a digest of the 33-mer were searched against an all gluten database. This yielded only the identification of *bona fide* peptides contained within the 33-mer (data not shown).

### pH activity profile of gliadin-degrading enzymes

Dental plaque bacteria suspended in 2 ml milliQ water were sonicated using a Branson Sonifier 450 at an output setting of 5 (30 Watts). The sample was sonicated for 30 cycles of 10 seconds each and was chilled on ice to prevent temperature increase and protease inactivation. The supernatant was harvested by centrifugation for 2 min at 10,000× *g*, aliquoted into 8 equal portions and concentrated to ∼20 ul using a speedvac (Savant). Each of the fractions was subjected to gliadin zymography (see details below). After electrophoresis and renaturing of the gel, the 8 gel lanes were separated with a razor blade and developed in 20 mM TrisBase/TrisHCl solutions adjusted to pH 3, 4, 5, 6, 7, 8, 9 or 10, respectively.

### Liquid iso-electric focusing (IEF) of gliadin-degrading enzymes

A concentrated suspension of plaque bacteria in 2 ml milliQ water was sonicated as described above. The sample was centrifuged for 2 min at 10,000× *g* and the supernatant was diluted with water to a final volume of 3 ml (S1). The protein concentration in S1 was determined at 215 nm (ε = 20). A 300 µl aliquot of S1 was set aside. To the remainder of the solution, a mixture of ampholytes (Bio-lyte 3/10, Bio-Rad, Hercules, CA) were added to a final concentration of 5% (v/v) (S2). The electrode assemblies of a MicroRotofor focusing chamber (Bio-Rad) were filled with 0.1 M phosphoric acid (anode electrolyte) and 0.1 M sodium hydroxide (cathode electrolyte). S2 was applied to the separating chamber using a syringe and native liquid IEF was performed at 4°C at 1 W applying a gradual increase in voltage from 50 V to 320 V over a 2 h time span. Ten 200 µl fractions (F1–F10) containing plaque supernatant proteins separated based on iso-electric point were harvested in a tray under vacuum according to the manufacturer's instructions. The pH value of each fraction was determined using pH strips. S1 and F1-F10 were subsequently concentrated to ∼20 ul using a speed-vac (Savant), and analyzed for protease activity by gliadin zymography.

### Gliadin zymography

Samples to be analyzed were suspended in sample buffer containing 0.25 M Tris/HCl, 10% glycerol, 2% SDS and 0.0025% bromophenol blue. The separating gel contained 375 mM Tris/HCl (pH 8.8), 8% acryl/bisacrylamide (19∶1 ratio), 0.1% (w/v) SDS, 2 mg/ml gliadin, 0.05% (w/v) ammonium persulfate (APS) and 0.04% (v/v) TEMED. The stacking gel contained 126 mM TrisHCl (pH 6.8), 4% acryl/bisacrylamide (19∶1), 0.1% (w/v) SDS, 0.05% (w/v) APS and 0.1% (v/v) TEMED. The running buffer contained 24 mM TrisBase, 192 mM glycine and 0.1% (w/v) SDS (pH 8.3). Electrophoresis was carried out at a constant voltage of 100 V at 4°C. Gels were renatured by washing for 2×30 min at 22°C in zymogram renaturing buffer followed by washing for 2×20 min in zymogram developing buffer (both obtained from InVitrogen) and incubation in the same buffer for 48 h. Gels were stained in 40% methanol/10%acetic acid/0.1% Coomassie brilliant blue R-250 and destained in the same solution without dye.

### Alignment of gliadin epitopes with basic PRPs

Sequences of 10 T-cell stimulatory peptides [21] were aligned with basic PRP1, 2, 3, and 4 using the Sim alignment tool at the ExPASy proteomics server and ClustalX software (Version 2.0.12) applying the following parameters: comparison matrix: BLOSUM62; gap open penalty: 12; gap extension penalty: 4.

## Results

### Structural similarities between gliadins and basic PRPs

The recent discovery of an oral glutamine endoprotease cleaving basic PRPs prompted us to study their capability to cleave immunogenic epitopes of gliadins which drive intestinal inflammation in celiac disease. When the primary amino acid sequences of gliadins and basic PRPs were compared, both protein families were found to share interesting structural features. First, gliadins and basic PRPs are very similar in size, averaging 307 and 357 amino acids (including signal peptides), respectively. Second, in gliadins as well as basic PRPs the amino acids glutamine (Q) and proline (P) combined account for about 50% of the total amino acid composition (Table 1). A noticeable difference though is that the relative proportion of glutamine and prolines in gliadins and basic PRPs is reversed. Third, the XPQ sequence, prevalent in basic PRPs is also frequently occurring in gliadins. An example of a comparison between the amino acid sequences of a basic PRP2 and ω-5 gliadin is shown in Table 2. In some gliadins such as ω-5 gliadin, the XPQ tripeptide is present at an exceptionally high frequency, even exceeding that observed in basic PRBs (Table 1).

**Table 1:** Characteristics of gliadins from *Triticum aestivum* and human salivary basic proline-rich proteins.^1^

Protein	# a.a^a^	%Q	%P	%(Q+P)	#XPQ
α/β-gliadins	288^b^	34	15	49	7–22
γ-gliadins	276^c^	31	16	47	2–38
ω-gliadins	356^d^	24	19	43	8–72
PRB1	392	16	37	53	47
PRB2	416	15	37	52	50
PRB3	309	14	35	49	20
PRB4	310	14	34	48	21

a Including signal peptides.

b,c,d Average number of amino acids in α/β-gliadins (58 entries),

γ-gliadins (110 entries), ω-gliadins (8 entries).

**Table 2:** Comparison of amino acid sequences of salivary basic proline-rich protein 2 (PRB2) from human saliva and wheat omega-5 protein from *Triticum aestivum*.*

>sp|P02812|PRB2_HUMAN Basic salivary proline-rich protein 2 OS = Homo sapiens GN = PRB2 PE = 1 SV = 3 MLLILLSVALLALSSAQNLNEDVSQEESPSLIAGNPQGAPPQGGNKPQGPPSPPGKPQGP PPQGGNQPQGPPPPPGKPQGPPPQGGNKPQGPPPPGKPQGPPPQGDKSRSPRSPPGKPQG PPPQGGNQPQGPPPPPGKPQGPPPQGGNKPQGPPPPGKPQGPPPQGDNKSRSSRSPPGKP QGPPPQGGNQPQGPPPPPGKPQGPPPQGGNKPQGPPPPGKPQGPPPQGDNKSQSARSPPG KPQGPPPQGGNQPQGPPPPPGKPQGPPPQGGNKSQGPPPPGKPQGPPPQGGSKSRSSRSP PGKPQGPPPQGGNQPQGPPPPPGKPQGPPPQGGNKPQGPPPPGKPQGPPPQGGSKSRSAR SPPGKPQGPPQQEGNNPQGPPPPAGGNPQQPQAPPAGQPQGPPRPPQGGRPSRPPQ
>tr|Q402I5|Q402I5_WHEAT Omega-5 gliadin OS = Triticum aestivum PE = 4 SV = 1 MKTFIIFVLLAMAMNIASASRLLSPRGKELHTPQEQFPQQQQFPQPQQFPQQQIPQQHQI PQQPQQFPQQQQFLQQQQIPQQQIPQQHQIPQQPQQFPQQQQFPQQHQSPQQQFPQQQFP QQKLPQQEFPQQQISQQPQQLPQQQQIPQQPQQFLQQQQFPQQQPPQQHQFPQQQLPQQQ QIPQQQQIPQQPQQIPQQQQIPQQPQQFPQQQFPQQQFPQQQFPQQEFPQQQQFPQQQIA RQPQQLPQQQQIPQQPQQFPQQQQFPQQQSPQQQQFPQQQFPQQQQLPQKQFPQPQQIPQ QQQIPQQPQQFPQQQFPQQQQFPQQQEFPQQQFPQQQFHQQQLPQQQFPQQQFPQQQFPQ QQQFPQQQQLTQQQFPRPQQSPEQQQFPQQQFPQQPPQQFPQQQFPIPYPPQQSEEPSPY QQYPQQQPSGSDVISISGL

*XPQ sequences are underlined.

### Hydrolysis of gliadin-derived synthetic enzyme substrates

**Figure 1:**
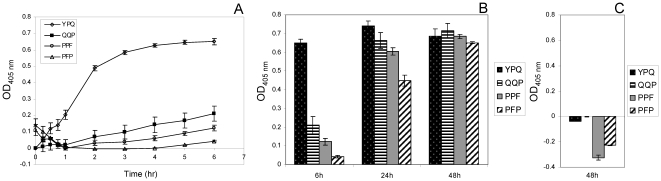
Hydrolysis of gliadin-derived enzymatic substrates. Dental plaque was suspended in saliva ion buffer to an OD_620_ of 1.2. Substrates Z-YPQ-pNA, Z-QQP-pNA, Z-PPF-pNA and Z-PFP-pNA were added to final concentrations of 200 µM. Substrate conversion was monitored spectrophotometrically at 405 nm. A, hydrolysis measured during the 0–6 hr time interval; B, hydrolysis measured after 6 h, 24 h and 48 h; C, substrates incubated for 48 h in saliva ion buffer only (control).

The KPQ tripeptide is abundant in salivary basic proline-rich proteins and cleaved effectively [14]. While KPQ is not present in gliadins, YPQ is a relatively abundant tripeptide, especially in the immunogenic 33-mer peptide. To study oral microbial enzyme specificities directed at gliadins, we synthesized Z-YPQ-pNA, as well as three other gliadin-derived tripeptide analogs, Z-QQP-pNA, Z-PPF-pNA and Z-PFP-pNA with Z representing benzyloxycarbonyl (protective group) and pNA representing paranitroanilide (reporter group). The substrates were incubated with a suspension of plaque bacteria and hydrolysis was monitored spectrophotometrically. Among the four substrates, Z-YPQ-pNA was most efficiently cleaved by enzymes contained in dental plaque (Figure 1A). However, all four substrates were hydrolyzed at extended incubation times (Figure 1B). No cleavage was noted when substrates were incubated in saliva ion buffer only (Figure 1C), even negative values were observed which can be explained by resolution of minor precipitates as the incubation time progressed. Likewise, no substrate hydrolysis was observed in the presence of trypsin (data not shown). The diverse cleavage specificities associated with plaque bacteria suggests the presence of multiple proteolytic enzymes capable of targeting dietary gliadins.

### Degradation of gliadins

**Figure 2:**
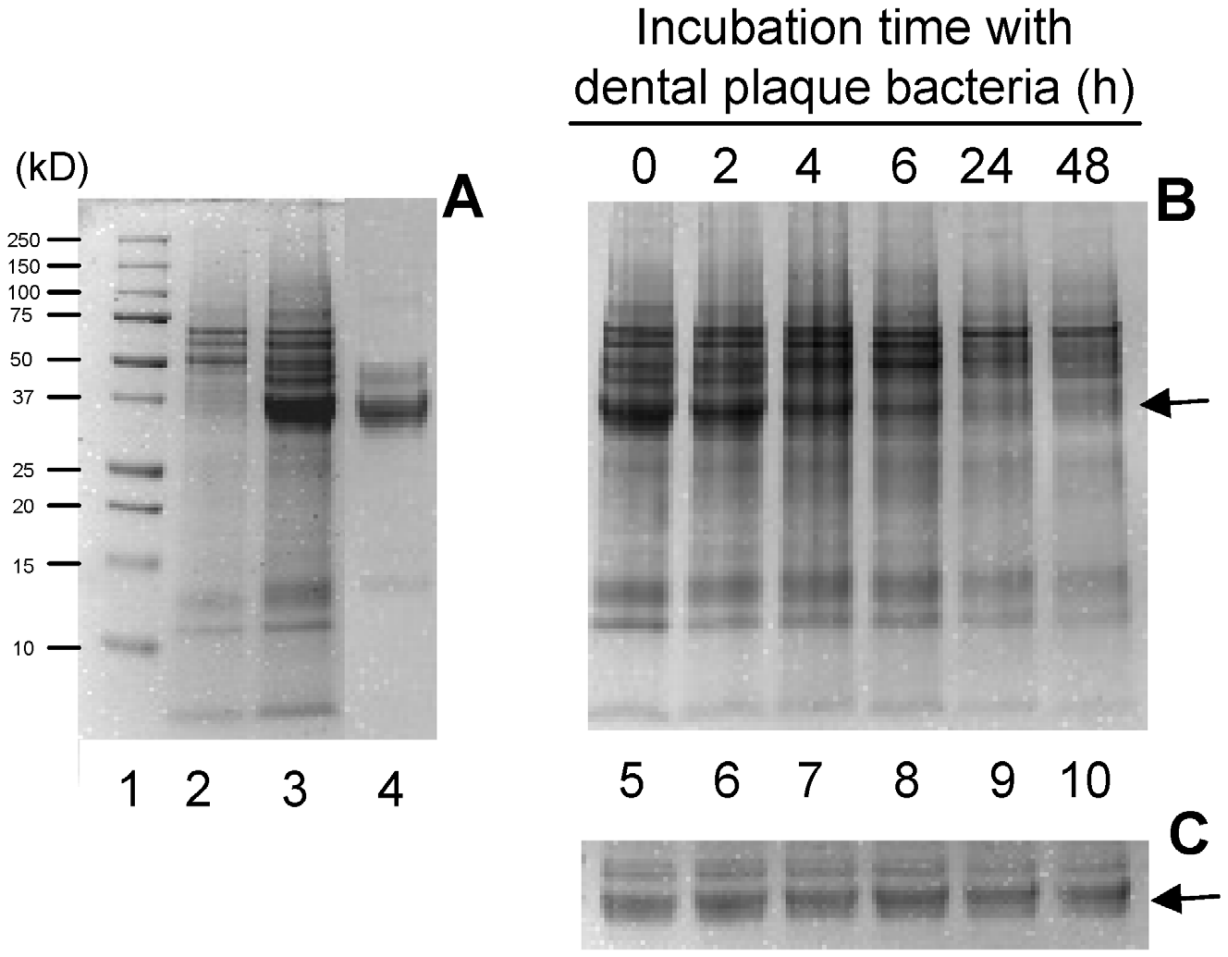
Degradation of a mixture of gliadins (G) by oral plaque microorganisms. Dental plaque was suspended in saliva ion buffer to an OD_620_ of 1.2. Gliadin (Sigma) was added to a final concentration of 250 µg/ml. After various incubation times at 37°C 100 µl aliquots were removed, boiled and analyzed by SDS PAGE. A, lane 1, Molecular weight standard; lane 2: plaque bacterial suspension only (t = 0); lane 3: plaque suspension + gliadin (t = 0); lane 4: 25 µg gliadin (t = 0); B, lanes 5 to 10: plaque suspension + gliadin, sampled after t = 0, t = 2 h, t = 4 h, t = 6 h, t = 24 h and t = 48 h, respectively. C, gliadin incubated for the same time intervals in saliva buffer only (control).

To investigate if gliadins were cleaved by oral microbial proteases, a commercial preparation of a mixture of all gliadins was used, containing a variety of α/β-, ω- and γ-gliadins. Gliadins stain poorly with coomassie and appeared as major bands in the ∼35–47 kD region (Figure 2, lane 4). Other minor components, including traces of albumins, globulins and glutenins, may also be present but were likely low in content. Our studies focused on the protease sensitivity of the ∼35–47 kD protein bands. Figure 2 shows that after ∼6 h incubation with plaque bacteria the 35–47 kD bands had undergone substantial degradation and were virtually undetectable after 24 h incubation whereas gliadins were stable in saliva ion buffer only.

### Proteolysis of synthetic gliadin peptides (33-mer and 26-mer)

**Figure 3:**
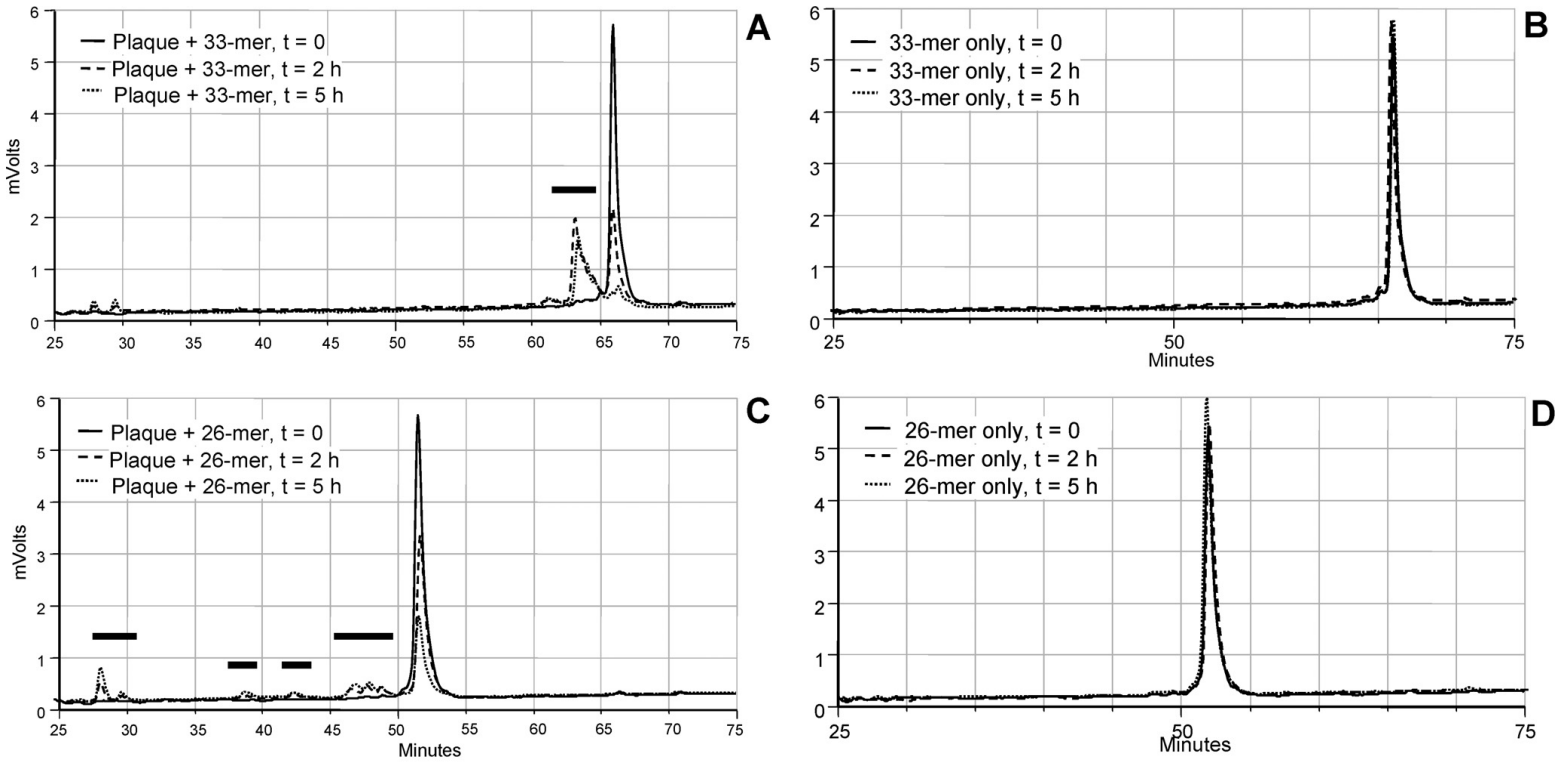
Degradation of gliadin-derived peptides by dental plaque bacteria. The 33-mer or the 26-mer were added to a final concentration of 250 µg/ml to a suspension of dental plaque bacteria in saliva ion buffer (OD_620_ 1.2). After t = 0 h, 2 h and 5 h, 100 µl aliquots were removed, boiled and subjected to RP-HPLC. A and C, chromatograms of the 33-mer and 26-mer peptides incubated in dental plaque suspension; B and D, 33-mer and 26-mer peptides incubated in saliva ion buffer only (control).

Within the gliadin sequences, certain peptide regions are particularly immunogenic and resistant to degradation by human-derived digestive enzymes. These are a 33-mer peptide present in α2-gliadin, also denoted as “superantigen” [13], [17], [22], and a 26-mer domain derived from γ-gliadin[18]. The susceptibility of these peptides to proteolytic breakdown by oral microbial enzymes was investigated by RP-HPLC. The intact 33-mer peptide at the applied gradient eluted after 66 min (Figure 3A). We confirmed that both peptides are resistant to digestion by the major human digestive enzymes trypsin, chymotrypsin and pepsin after 24 h of incubation (data not shown). In a suspension of plaque bacteria, however, the 33-mer completely degraded after 5 h of incubation, as evidenced by disappearance of the peak at 66 min and appearance of degradation fragments eluting between 60–66 minutes and between 25 and 30 minutes. The 26-mer was also cleaved by dental plaque bacteria (Figure 3C), yielding fragments eluting between 25 and 30 minutes and between 35 minutes and 52 minutes. No degradation of the 33-mer and the 26-mer was observed when incubations were carried out in saliva ion buffer alone (Figures 3B and 3D, respectively), indicating that the peptides were stable unless oral bacterial enzymes were present in the incubation mixture.

**Figure 4:**
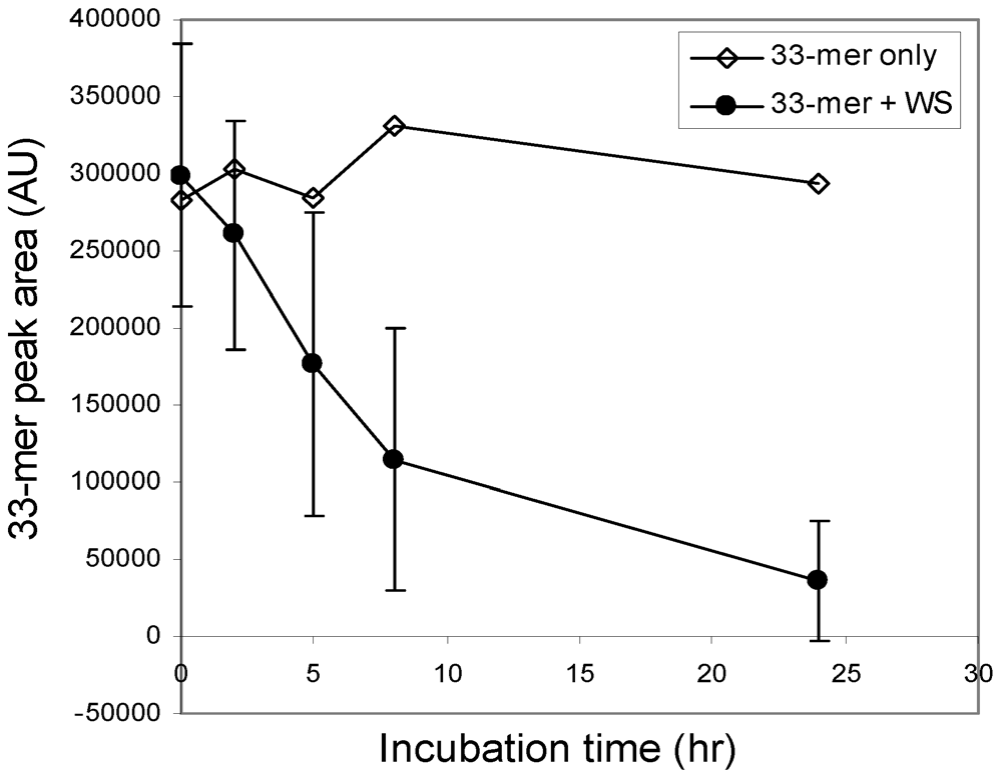
Degradation of the 33-mer in human whole saliva (WS). The 33-mer was added to a final concentration of 250 µg/ml to WS collected from eight individual subjects. In each of the saliva samples, the degradation of the 33-mer was monitored. The average peak area and standard deviation of the 33-mer incubated for 0 h, 2 h, 5 h, 8 h and 24 h in the eight WS samples are shown. Control: 33-mer only, incubated in saliva ion buffer for the same time intervals.

In addition we investigated if protease activities are detectable in human WS. WS contains about 108 oral microorganisms per ml and is continuously swallowed reaching more downstream regions of the gastro-intestinal tract. Data showed that the 33-mer was proteolytically cleaved in WS from all eight healthy human subjects examined, but substantial variation in activities was noticeable between subjects (Figure 4).

### LC-ESI-MS/MS of fragments from the 33-mer and 26-mer

In order to anticipate if oral microbial enzymes could cleave and theoretically neutralize the immunogenic epitopes contained within the 33-mer 26-mer sequences, the cleavage products of both peptides were structurally characterized by LC-ESI-MS/MS. The RP-HPLC fractions collected for this purpose are indicated with horizontal bars in Figures 3A and 3C. The fragments generated from the 33-mer and the 26-mer are presented in supplemental Figure S1. Short N- and C-terminal peptides (<7 amino acids) were not detected, since these eluded detection by LC-ESI-MS/MS for technical reasons. The peptides identified with high confidence show that virtually all sites are targeted in both the 33-mer and the 26-mer. The diversity of peptides generated in mixed dental plaque is reflective of the multitude of enzymatic activities that can be found in this biofilm. Dental plaque furthermore likely contains exopeptidases that could have contributed to the pattern observed. While proteolysis did not abolish all immunogenic epitopes in the time span examined, the results together with hydrolysis of -pNA derivatized substrates indicate that the gliadins are excellent substrates for oral microbial enzymes.

### pH activity profile of oral gliadin-degrading enzymes

**Figure 5:**
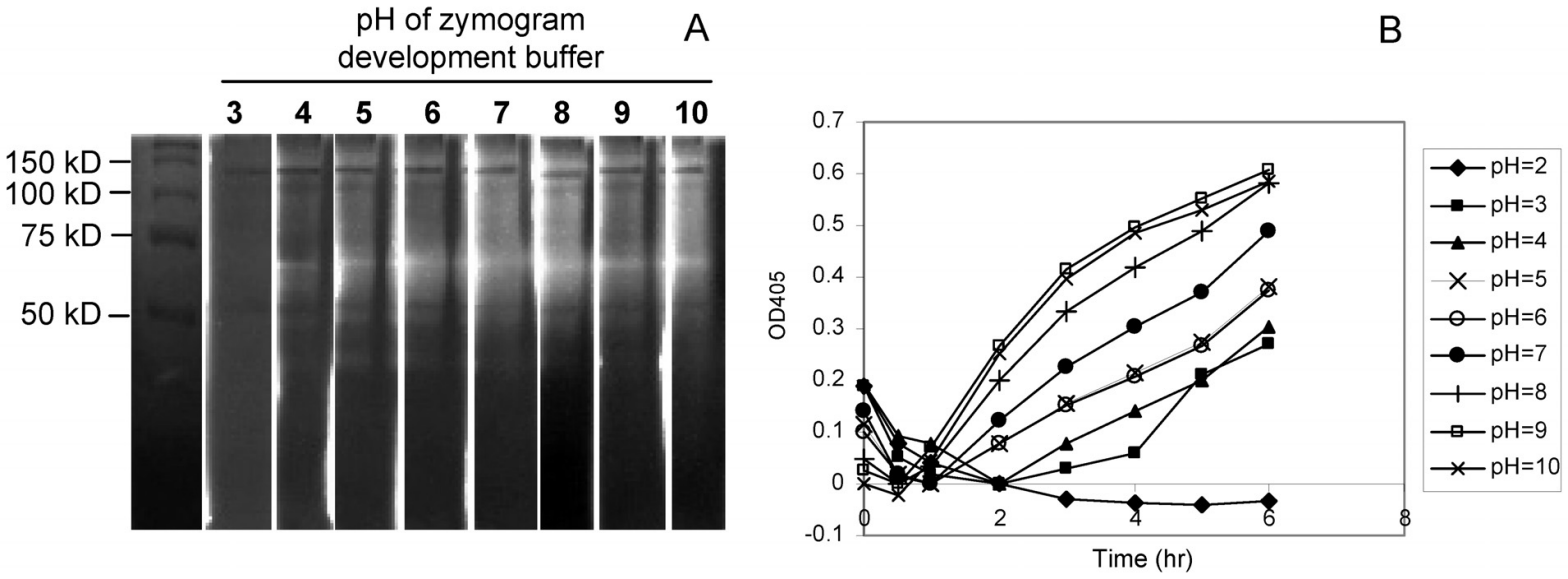
Determination of the pH activity profile of oral gliadin-degrading enzymes. A, Gliadin zymography. Sonicated plaque bacterial supernatant was aliquoted into eight equal fractions. Each fraction containing 90 µg protein was loaded onto a gliadin zymogram gel. After electrophoresis and renaturing the gel was cut and individual lanes were developed in 20 mM Tris buffers exhibiting pH values from 3 to 10. Far left lane: molecular weight standard. B, Z-YPQ-hydrolysis. Plaque bacteria were suspended 20 mM Tris buffers varying in pH from 2 to 10 to a final OD_620_ of 1.2. Suspensions were mixed with Z-YPQ-pNA (200 µM). Substrate hydrolysis was monitored spectrophotometrically at 405 nm. A representative graph of 3 independent experiments is shown.

In view of the varying pH conditions along the gastro-intestinal tract, the pH activity profile of the oral gliadin-degrading enzymes was investigated. For this purpose plaque was sonicated which released the enzymes into the supernatant. The supernatant fraction was subjected to gliadin zymography and developed in buffers with pH values ranging from 3 to 10 (Figure 5A). The major activity in the sonicate supernatant was associated with a band with an Mr ∼70 kD. Gliadin degradation was observed in gel strips that were developed in buffers with pH values from 4 to 10 with optimal gliadin-degrading activity at pH 8–10. Weak activity was observed at pH = 3. Activity at this pH was confirmed with the synthetic substrate Z-YPQ-pNA (Figure 5B). However, at pH = 2 no activity could be demonstrated.

### Overall iso-electric points of oral gliadin-degrading enzymes

**Figure 6:**
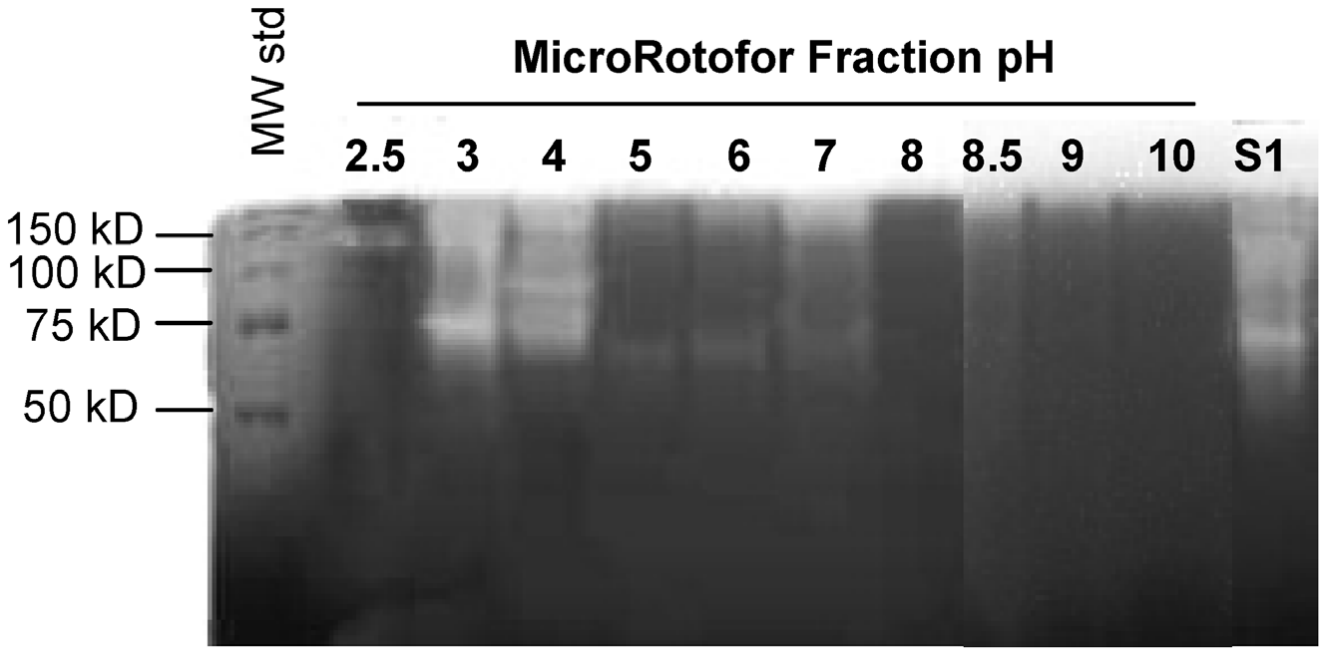
Isoelectric focusing profile of the gliadin degrading enzymes. Proteins in sonicated plaque bacteria supernatant were separated based on isoelectric point by liquid iso-electric focusing (IEF). The enzymatic activity in each of the 10 IEF fractions was established by gliadin zymography. Far left lane, MW standard; S1: plaque supernatant sample before IEF.

The pH activity experiment provided a first assessment of the overall molecular weights - and pH dependence- of the major proteolytic components in dental plaque. To investigate additional physicochemical properties of the enzymes of interest, we subjected the plaque supernatant protein mixture to liquid iso-electric focusing (IEF). The pH of the ten fractions collected ranged from 2.5 to 10. Protease activities in each of the fractions were then examined by gliadin zymography (Figure 6). Most of the proteases had migrated to the anode and appeared in fractions with pH values of 3–4. In the most acidic fraction (pH 2.5) a high molecular weight protease of Mr ∼140 kD was observed. In the pH 3.0 fraction enzymes with strong activity were noted of ∼70 kD as well as enzymes with higher Mr values, perhaps representing multimeric variants of the 70 kD enzyme. At pH 4.0 various enzymatic bands were observed in the same 70 kD Mr region. Weak protease activities of ∼65–70 kD were noted in fractions with pH values of 5, 6, and 7, and weak activities associated with higher Mr species (>150 kD) were discernable in fractions exhibiting alkaline pH values. Overall, the results indicate that the most prominent gliadin-degrading enzymes in the oral cavity are acidic in nature.

## Discussion

Between ∼0.5 and 2% of a major part of the human population has more or less severe forms of celiac disease [13]. Most of those diagnosed profit from a strict life-long gluten free diet which is extremely difficult to maintain, especially since even minor gluten contamination in foods which are omnipresent needs to be ruled out [23]. One of the therapeutic strategies to counteract or prevent the deleterious effect of these minor amounts of dietary gluten focuses on proteolytic enzymes to aid in their degradation, thus preventing their antigenic presentation to and activation of intestinal T cells [24], [25]. The results presented in this manuscript indicate that unexpectedly such potent digestive enzymes are naturally associated with the upper GI tract, i.e., the oral cavity. Our studies on oral microbial proteases showed that: 1) the tripeptide YPQ which frequently occurs in gluten is preferentially targeted; 2) gliadin as well as two otherwise highly immunogenic and protease-resistant gliadin peptides are proteolytically degraded; 3) gliadin-degrading enzymes are active over a wide pH range (pH 3–10); and 4) most of the oral gliadin-degrading enzymes are acidic in nature, exhibiting IEP values of 2.5 to 4. The data presented suggest a potential role for the oral microbiome in gluten digestion. Furthermore, given the broad pH activity range and partial species overlap between oral, oesophageal and duodenal GI microbiomes, it is likely that gliadin-degrading activities extend well beyond the oral region.

While novel with respect to source, gliadin degrading enzymes have been discovered in nature and are being exploited for their potential therapeutic application as dietary supplements in celiac disease. One of the enzymes that is being considered for this purpose is EP-B2 which is a glutamine endoprotease produced from germinating wheat [10]. Another group of enzymes capable of cleaving gluten are the prolyl endoproteases (PEP), specifically, PEP from *Aspergillus niger* (AN-PEP) [26] and PEP from *Sphingomonas capsulata* (SC-PEP) [27]. EP-B2, AN-PEP and SC-PEP have been studied as food additives, either alone or in combination [28] and are in clinical phase I and IIa trials (Clinicaltrails.gov identifiers NCT00810654 and NCT00959114). An important difference between these enzymes and those found in the GI tract is the fact that the former enzymes originate from non-human associated sources, i.e. non-resident bacteria or plants. Resident gluten-degrading microorganisms described in the present manuscript are a viable source of novel enzyme(s) and offer the additional advantage to be potentially exploited as probiotic agents to generate more long lasting changes in GI gluten digestive capacity.

For the reasons pointed out above it will be of high interest to obtain the individual enzymes originating from oral bacterial sources that are cleaving gliadin. Human saliva contains >600 different microbial strains and careful selection strategies need to be applied to identify the most active organism(s). Studies in our laboratory are underway to identify and characterize which microorganisms in this microbial mixture exhibit gliadin degrading capacities. We have already identified four bacterial strains with cleavage activities far exceeding those of the mixture of bacteria that can be found in dental plaque (manuscript in preparation). Identification of such bacteria is a first step towards the isolation, cloning and full characterization of the gliadin-degrading enzymes, and subsequent evaluation of these enzymes in *in vitro* and *in vivo* gliadin detoxification assays.

Human saliva-derived basic PRPs and wheat-derived gliadins show noticeable similarities in their amino acid composition with proline and glutamine being the chief constituent amino acids. Since gliadins contain well defined T-cell stimulatory domains for patients carrying HLA-DQ2 or HLA–DQ8 [21], [29], [30] we studied to what extent these gliadin domains show structural overlap with salivary basic PRPs. While it would be highly unlikely that the exact immunogenic domains stimulating T cell in patients with celiac disease are present in basic PRPs, given the successful reversal of celiac symptoms by gluten elimination from the diet in most patients, it is of interest that some celiac patients are refractory to a gluten-free diet [31]. The major basic salivary proline-rich proteins are encoded by *PRB1*, *PRB2*, *PRB3* and *PRB4* and contain multiple repeat domains of 20 or 21 amino acids [32] (supplemental Figure S2). The alignment of gliadin epitopes [33] with selected regions in the basic PRP sequences is depicted in supplemental Figure S3. The comparison revealed that gliadin epitopes are up to 50% homologous with basic PRP sequences. Due to the repeat domain structures in basic PRPs the gliadin-like domains are in some cases present in multiple copies. While the percentage overlap is substantial, it is evident from this analysis that no single point mutation in a basic PRP gene would generate a classical toxic gliadin epitope. Nevertheless, given the noted structural resemblances between these two protein families, and the fact that T-cell epitopes in gliadins typically cluster in regions with a high proline content [34], it will be of interest to characterize the salivary PRP geno- and phenotype of celiac patients.

There must be a biological reason, perhaps a nutritional reason that human saliva contains proteins that are so similar in composition to dietary proteins. Both protein families enter the GI tract and come into contact with resident GI microorganisms. Per day about 0.8 L of saliva is produced, primarily by the major salivary glands, and swallowed. Of note, salivary PRPs constitute approximately 70% of all secreted salivary proteins [35]. With an average WS protein concentration of 2 mg/ml of protein, it can be estimated that about 1 g of PRPs and derived peptides enter the GI system each day. In addition, the Western daily diet contains about 10–20 g of gluten proteins, which are cleaved in part and incompletely by human digestive enzymes and, as we hypothesize, are further fragmented by GI microbial proteases. The high numbers of proline/glutamine containing peptides from different sources continuously entering the gastro-intestinal lumen is an intriguing notion. In addition, glutamine endoprotease-producing microorganisms are also constantly being swallowed with saliva. It may be speculated that proline/glutamine peptide profiles and/or GI microbial glutamine endoprotease activities show differences between healthy and celiac patients, which could be important clinically.

Finally, our results lay the foundation for a further characterization of the oral bacteria that secrete gliadin (gluten) degrading enzymes as well as identification and purification of their most active gliadin-cleaving enzymes. Apart from interesting biological findings, these bacteria and enzymes may lead to novel and effective strategies to detoxify immunogenic gluten peptides prior to reaching the proximal small intestine.

## Supporting Information

Figure S1 Fragmentation of gliadin 33-mer and 26-mer by oral microbial enzymes in dental plaque. The 33-mer and the 26-mer were added to plaque suspension in saliva ion buffer (OD620 1.2) to a final concentration of 250 µg/ml and incubated for 5 h. Samples were analyzed by RP-HPLC, degradation fragments were collected and subjected to LC-ESI-MS/MS. The peptides identified in the individual RP-HPLC fractions were combined.
(4.23 MB TIF)

Figure S2 Schematic presentation of the structures of human salivary basic proline-rich proteins. White boxed areas: signal peptides; dark grey boxed areas: non-repeat domains; light grey boxed areas: repeat domains. The consensus amino acid sequences of the repeat domains are indicated. In PRB1L and PRB2L some of the repeat domains are interspaced with single proline (P) or serine (S) residues. Open circles: repeat regions missing in the truncated M-allelic isoforms; diamonds: repeat regions missing in the truncated S-allelic isoforms. Note in PRB4 that the missing segments in the M and S isoforms overlap only in part with the repeat domains.
(0.21 MB TIF)

Figure S3 Sequence similarities between salivary basic proline-rich proteins and immunotoxic gliadin epitopes. Displayed are selected regions within the basic PRP structures (top sequences, bolded) and gliadin epitopes (underneath). The amino acids that are homologous in the compared sequences are indicated with an asterisk.
(4.23 MB TIF)
