# Supplementary figures and images for: Discovery of a Novel and Rich Source of Gluten-Degrading Microbial Enzymes in the Oral Cavity
Source: PLoS One. 2010 Oct 11;5(10):e13264. doi: 10.1371/journal.pone.0013264 (PMC2952584; doi:10.1371/journal.pone.0013264)

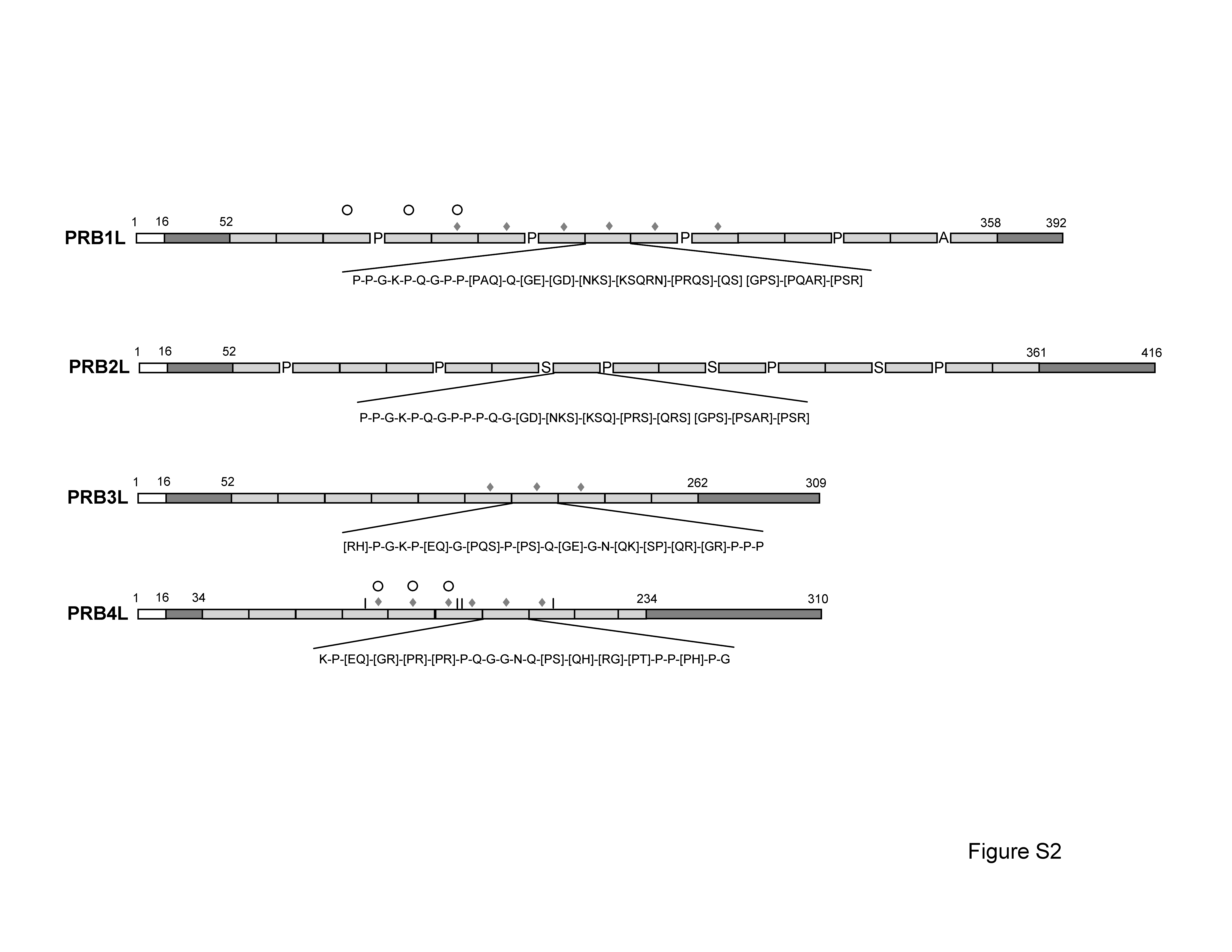

Supplement: Figure S2 — Schematic presentation of the structures of human salivary basic proline-rich proteins. White boxed areas: signal peptides; dark grey boxed areas: non-repeat domains; light grey boxed areas: repeat domains. The consensus amino acid sequences of the repeat domains are indicated. In PRB1L and PRB2L some of the repeat domains are interspaced with single proline (P) or serine (S) residues. Open circles: repeat regions missing in the truncated M-allelic isoforms; diamonds: repeat regions missing in the truncated S-allelic isoforms. Note in PRB4 that the missing segments in the M and S isoforms overlap only in part with the repeat domains. (0.21 MB TIF) [file pone.0013264.s002.tif]
